# Editorial: Insights and advances in surgical best practices for gender health equity and appropriateness

**DOI:** 10.3389/fsurg.2025.1657078

**Published:** 2025-07-11

**Authors:** Sara Sablone, Pietro Refolo, Rossana Cecchi

**Affiliations:** ^1^ Section of Legal Medicine, Interdisciplinary Department of Medicine, Policlinico-University of Bari “Aldo Moro”, Bari, Italy; ^2^ Section of Bioethics and Medical Humanities, Department of Health Care Surveillance and Bioethics, Università Cattolica del Sacro Cuore, Rome, Italy; ^3^ Department of Biomedical, Metabolic and Neuroscience, University of Modena and Reggio Emilia, Modena, Italy

**Keywords:** gender medicine, personalized medicine, patient-centered care, health equity, appropriateness, ethics

**Editorial on the Research Topic**
Insights and advances in surgical best practices for gender health equity and appropriateness

Gender health equity in surgical practices is a rapidly evolving area of research in the field of personalized medicine, emerging as a pivotal innovation in healthcare and leveraging data to tailor diagnostics, prognostics, and treatments to individual patients.

The concept of individual variability, highlighted by Osler since the late 19th century, became more evident with the discovery of blood groups in the early 20th century and deepened in the 1950s through studies on anomalous drug responses. This progression has led to modern developments such as pharmacogenetics and gender medicine and laid the foundation for the current shift from a “one-size-fits-all” paradigm to more individualized models of healthcare, highlighting the importance of gender medicine in the development of personalized and patient-centered care.

The World Health Organization's emphasis on this matter has recognized gender-based approaches as the only reliable responses to gender-based disparities affecting the right to health and the need for fully inclusive personalized medicine.

However, there is still a significant gap in understanding how patients' sex-based biological factors (e.g., hormonal influences, chromosomal differences) and gender-related variables (e.g., behaviors, roles, and sociocultural aspects) can impact the complexity of care and disease load, an issue with both ethical and economic implications. Indeed, gender's influence on health and disease remains underappreciated in medical and surgical interventions, producing disparities in healthcare delivery and impacting treatment safety and efficacy for both males and females.

With the primary aim of exploring the current state of gender-based and patient-centered approaches in surgically relevant pathologies, focusing on specific preventive and diagnostic protocols, treatments, techniques, and outcomes, and investigating gender-related stratification in epidemiological analysis of surgical interest, we showcase the selected articles published in this Research Topic.

Capasso et al. performed a narrative review on the physiological causes of gender inequities in medical training and practice. Notably, they explored the unique challenges that young female residents face during pregnancy by focusing on the medical residency programs of obstetrics & gynecology and forensic pathology. Through a relevant regulatory framework analysis, the authors highlight the need for targeted support and provide suggestions for innovative solutions (such as augmented reality and metaverse) to mitigate biological risks and enable pregnant surgeons to receive continuous training during this topical lifetime without a disadvantageous learning curve conditioning compared to male colleagues.

Shao et al. suggested a gender-specific innovative approach to enhance surgical effectiveness and achieve better surgical outcomes for female patients undergoing gynecological surgery. They documented the initial experience by using methylene blue to label ureters in gynecological laparoscopic procedures and to prevent iatrogenic injuries.

Zhang et al. provided valuable insights into the relationship between socioeconomic status (SES), surgery delay, and survival outcomes in patients with non-metastatic papillary thyroid cancer (PTC). By emphasizing the importance of individualized treatments, their findings suggest that surgery for non-metastatic PTC can be delayed safely without a significant increase in mortality. However, disparities in healthcare access and broader socioeconomic inequities remain evident, as populations with lower SES show elevated non-PTC mortality. Simultaneously, better overall survival can be recorded among patients with independent predictors (such as those receiving neck dissection or radioactive iodine therapy, being married, and living in urban, higher-income areas), underscoring the interplay of sociodemographic and clinical factors. These findings call for targeted interventions to address healthcare disparities, particularly for vulnerable populations, including unmarried, older, rural, or minority patients.

Chen et al. conducted a study to estimate the median effective dose (ED50) of remimazolam combined with different butorphanol doses on the sedative effect for first-trimester artificial abortion, a short but painful and uncomfortable procedure. As the optimal dose of remimazolam plus butorphanol for sedation during a painless abortion is unknown, the effects of different butorphanol doses on the remimazolam ED50 in inhibiting the response of cervical dilatation were investigated. Thus, the authors provided a reference for drug safety and rational use in painless artificial abortion.

Ju et al. performed a meta-analysis of observational studies to verify the possible correlation between intraoperative hypothermia and surgical site infections (SSI). They excluded any connection and recognized the need for more randomized controlled trials to determine the role of intraoperative hypothermia in the SSI development in high-risk surgical procedures and patient populations.

Overall, this valuable article selection highlights that, in the field of personalized and gender medicine, one of the most pressing issues concerns the redefinition of the standard of care. Indeed, the integration of personalized and gender-specific approaches requires a fundamental rethinking of clinical protocols, as traditional legal models rely on guidelines based on population averages while personalized medicine challenges these norms by promoting stratified pathways.

Despite the European Community's call for including gender medicine in university medical science curricula and the release of several master's degrees dedicated to this topic, these aspects are still underrepresented in medical curricula, clinical trials, and regulatory frameworks.

Instead, health systems should prepare for economic and infrastructural impacts of personalized therapies, often requiring advanced diagnostic tools, innovative surgical technique implementation, digital data management, and ongoing physician and surgeon education and training. Ensuring equal access to these innovations should be a priority issue in public health.

The convergence of fully inclusive personalized medicine represents a frontier in precision healthcare. By acknowledging individual variability at both molecular and sociocultural levels, this approach has the potential to enhance diagnostic accuracy, treatment efficacy, assessment of healthcare needs and experiences, and overall patient safety. However, realizing these potential requires an interdisciplinary collaboration and approach and inclusive research models that consider sex and gender as integral dimensions of medical-surgical sciences and practices.

The world is demanding greater equity and appropriateness in the resources distribution and gender medicine aligns with this important goal. Identifying the most effective interventions produces benefits not only for the patient in terms of psychophysical health but also for the community by optimizing healthcare resources. Therefore, when implementing any preventive, diagnostic, therapeutic, or rehabilitative activity, one must consider the patients’ identifying and individualizing aspects. It is a socio-economic imperative and an ethical and moral duty to the well-being of individuals and the entire human community.

